# Characteristics of Trauma Mortality in Patients with Aortic Injury in Harris County, Texas

**DOI:** 10.3390/jcm9092965

**Published:** 2020-09-14

**Authors:** Ronald Chang, Stacy A. Drake, John B. Holcomb, Garrett Phillips, Charles E. Wade, Kristofer M. Charlton-Ouw

**Affiliations:** 1 Division of Vascular Surgery, Emory University, Atlanta, GA 30322, USA; ronald.chang@emory.edu; 2 Center for Translational Injury Research, The University of Texas Health Science Center, Houston, TX 77030, USA; charles.e.wade@uth.tmc.edu; 3 College of Nursing, Texas A&M University, Houston, TX 77030, USA; sadrake@tamu.edu; 4 Department of Surgery, University of Alabama at Birmingham, Birmingham, AL 35294, USA; jbholcomb@uabmc.edu; 5 Harris County Institute of Forensic Sciences, Houston, TX 77030, USA; garrett.phillips@ifs.hctx.net; 6 Department of Surgery, McGovern Medical School, Houston, TX 77030, USA; 7 HCA Houston Healthcare, Gulf Coast Division, Houston, TX 77004, USA

**Keywords:** aortic injury, non-compressible torso hemorrhage, trauma

## Abstract

Background: The National Academies of Science have issued a call for zero preventable trauma deaths. The mortality characteristics in all patients with aortic injury are not well described. Methods: All prehospital and hospital medical examiner records for deaths occurring in Harris County, Texas in 2014 were retrospectively reviewed, and patients with traumatic aortic injury were selected. The level of aortic injury was categorized by zone (0 through 9) and further grouped by aortic region (arch, zones 0 to 2; descending thoracic, zones 3 to 5; visceral abdominal, zones 6 to 8; infrarenal, zone 9). Multiple investigators used standardized criteria to categorize deaths as preventable, potentially preventable, or non-preventable. Results: Of 1848 trauma deaths, 192 (10%) had aortic injury. There were 59 (31%) aortic arch, 144 (75%) descending thoracic, 19 (10%) visceral abdominal, and 20 (10%) infrarenal aortic injuries. There were 178 (93%) non-preventable deaths and 14 (7%) potentially preventable deaths, and none were preventable. Non-preventable deaths were associated with blunt trauma (69%) and the arch or thoracic aorta (93%), whereas potentially preventable deaths were associated with penetrating trauma (93%) and the visceral abdominal or infrarenal aorta (79%) (all *p* < 0.05). Half of potentially preventable deaths (*n* = 7) occurred at the scene, and half occurred at a trauma center. Conclusion: Potentially preventable deaths after aortic injury were associated with penetrating mechanism and injury to the visceral abdominal and/or infrarenal aorta. Optimal prehospital and ED treatment include temporizing hemorrhage control, hemostatic resuscitation, and faster transport to definitive treatment.

## 1. Introduction

Hemorrhage is a leading cause of preventable trauma death [1,2]. A retrospective study by Eastridge et al., of 4596 combat casualty mortalities from 2001 to 2011 reported that 24% of deaths were potentially preventable, of which 90% were related to inadequate hemorrhage control [3]. A National Academies of Sciences Report in 2016 estimated the civilian preventable trauma death rate to be 20%, or 30,000 deaths per year, and called for zero preventable trauma deaths [4]. This report discussed the military’s use of near 100% autopsy rate to identify strategies where targeted interventions could improve trauma patient outcomes. For example, Eastridge et al., reported decreased exsanguination rate due to peripheral hemorrhage by 85% (from 23.3 to 3.5 deaths per year) after implementation of increased military tourniquet use that resulted from a review of military autopsy data [3]. Translation of tourniquet use to the civilian sector in the form of the Stop the Bleed Campaign has also yielded positive results [5,6]. Recently, Drake et al. conducted a retrospective review of all death-related (including prehospital and hospital medical examiner) records that occurred during 2014 in Harris County, Texas and determined the preventable and potentially preventable trauma death rate to be 36% [7]. Although great progress has been made in control of peripheral hemorrhage, truncal hemorrhage (e.g., from aortic injury) remains a significant cause of trauma mortality [8]. Similar to the military’s methodology, the objective of the present study was to describe the anatomic characteristics and preventability of aortic injuries in deceased civilian trauma patients to identify potential targets for intervention.

## 2. Methods

**Figure 1:**
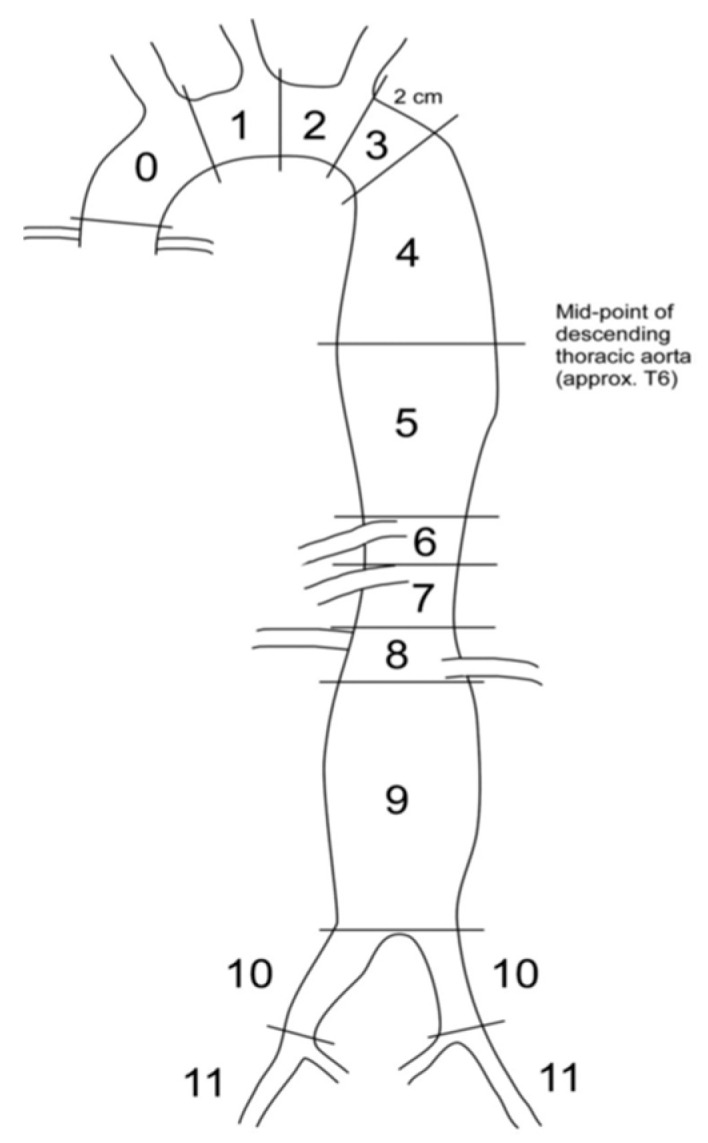
Zones of aortic injury [12].

This is a secondary analysis of a study by Drake et al. that examined all death-related records in 2014 in Harris County, TX [7], the third most populous county in the United States (4.4 million), which includes the Greater Houston area. Drake et al., included 1848 trauma deaths, of which 70% had an autopsy and the remaining 30% had extensive radiographic records. Each case was assigned a preventability level (preventable, potentially preventable, and non-preventable) by multidisciplinary review based on anatomic findings predominantly at autopsy. We defined preventable as clearly survivable anatomic injuries if appropriate steps had been taken (including deaths due to deviations from standard of care), potentially preventable as severe anatomic injuries that could be survivable with optimal care, and non-survivable as death due to anatomic injuries. Examples of non-survivable anatomic injuries include catastrophic brain injury (transcranial penetrating injury, brainstem disruption, etc.); atlanto-occipital dissociation; proximal cervical spinal cord transection; intra-thoracic airway transection; significant transmural lacerations to the heart, aorta, or main pulmonary artery with free hemorrhage; complete liver disruption; and catastrophic abdominopelvic injury (e.g., traumatic hemipelvectomy) [7]. Disagreements regarding preventability level were resolved by consensus according to an established protocol [9]. Causes of death were determined by the medical examiner and defined as the physiologic derangement leading to death [10]. Additional details regarding the study methods have been published previously [11]. Importantly, we included all deaths under the jurisdiction of the Harris County Medical Examiner, including prehospital deaths and those occurring in non-trauma centers, in addition to those occurring in trauma centers. The subset of patients with aortic injury was selected. Autopsy photographs and reports were reviewed by investigators. The location of aortic injury was categorized by zone (0 to 9) consistent with reporting standards for endovascular aortic treatment (Figure 1) [12]. Injuries in contiguous zones were treated as one injury. Those in non-contiguous zones were treated as multiple injuries. For each injury, the most proximal zone and the number of zones involved were recorded. To facilitate analyses, injuries were also categorized by aortic region (arch, zones 0 to 2; descending thoracic, zones 3 to 5; visceral abdominal, zones 6 to 8; and infrarenal, zone 9) with single injuries spanning multiple contiguous zones possibly involving multiple regions. This study was approved by the institutional review boards at both The University of Texas Health Science Center at Houston and Baylor College of Medicine (Houston, TX, USA). Informed consent was not required.

Stata 15.1 (Stata Corp., College Station, TX, USA) was used for all calculations. Data are summarized as median and interquartile range (IQR) or proportions as appropriate. Non-parametric univariate comparisons of continuous data were performed using the Wilcoxon rank-sum test. Comparisons of categorical data were performed with Chi-Squared test or Fisher’s exact test for categories with ≤5 expected cases. Statistical significance was defined as two-tailed alpha at 0.05 level.

## 3. Results

### 3.1. Demographics and Death Characteristics

Of 1848 trauma deaths in Harris County, TX in 2014, 192 (10.4%) had aortic injury, of which 191 (99%) had an autopsy. There were 178 (93%) non-preventable deaths (NP), 14 (7%) potentially preventable deaths (PP), and no preventable deaths. Demographics and death data are summarized in Table 1. There were no differences in age between NP and PP deaths. The incidence of blunt rather than penetrating trauma, death at the scene, and/or death with traumatic brain injury (TBI) were all more likely in the NP than PP groups (all *p* < 0.05).

### 3.2. Anatomic Description of Aortic Injuries

Aortic injuries as defined by most proximal involved zone are summarized in Table 2. There were no differences between NP and PP groups in the average number of zones involved per injury (1.46 vs. 1.31) and incidence of multiple injuries (13% vs. 14%) (both *p* > 0.05). Aortic injuries as defined by region are summarized in Table 3. NP deaths were more likely a result of injuries to the aortic arch or descending thoracic aorta, whereas PP deaths were more likely a result of injuries to the abdominal visceral or infrarenal aorta (all *p* < 0.05). Of 170 deaths who had any injuries to the arch or descending thoracic aorta, 166 (98%) were NP. Of 22 deaths who had aortic injuries confined to the visceral abdominal and/or infrarenal aorta, 10 (45%) were PP.

### 3.3. Subgroup Analysis of PP Deaths

PP deaths (*n* = 14) were examined in greater detail to better understand areas of potential improvement. Aortic injuries for seven patients who expired at the scene (Table 4) ranged from the descending thoracic aorta (zone 5) to the infrarenal aorta (zone 9). Of these, five had multiple zones injured and six sustained gunshot wounds and died of hemorrhage. The remaining seven patients survived to ED presentation (Table 5). All suffered from gunshot wounds, were transported to a trauma center by ground EMS, and had hemorrhage as the cause of death. None received prehospital transfusions as blood products were not available on ground EMS during the study period. The predominant zone of injury was the infrarenal aorta (zone 9). Three patients who presented to a level 3 trauma center were declared expired shortly after ED arrival. Four patients underwent resuscitative thoracotomy in the ED. Of these, three patients who presented to a level 1 trauma center underwent operative intervention for definitive hemorrhage control, of which two survived to ICU admission.

## 4. Discussion

We performed a secondary analysis of a study by Drake et al. which analyzed all trauma deaths that occurred in Harris County, TX in 2014. Of 192 cases with aortic injury, the autopsy rate was 99%, which allowed us to precisely describe the anatomic location of injury and categorize them by aortic zone and region. The majority of deaths were NP (93%), which were more often a result of blunt trauma and involved the thoracic aorta, while PP deaths (7%) were more often a result of penetrating trauma and involved the abdominal aorta. The maximum survival time after ED presentation was less than 9 h, there were no preventable deaths, and PP deaths were primarily due to hemorrhage. In contrast, Drake et al., reported an overall preventable/possibly preventable trauma (P/PP) death rate of 36%, with 43% of these dying after discharge from initial hospitalization [7]. Important causes of P/PP death in the overall study were hemorrhage and TBI initially, as well as sepsis, multiple organ failure, pulmonary embolism, and comorbid conditions after the initial period. Notably, the results of the present study are similar to those of a case series by Parmley et al., published in 1958 that analyzed 275 cases of blunt aortic injury and had >99% autopsy rate [13]. They reported 86% were dead on arrival, long-term (>1 year) survival of 2%, and aortic isthmus (zone 3) as the most common site of injury (37%). Comparatively, we report 81% dead at the scene, potentially preventable death rate of 7%, and the proximal descending thoracic aorta (zone 4) as the most common site of injury (31%). These similarities, which have persisted over six decades, are a testament to the gravity of this problem and the challenges in its prevention and treatment.

Diagnosis and management of traumatic aortic injuries have changed significantly over the last twenty years with the widespread use of computed tomography imaging for trauma and advent of endovascular treatment of aortic lesions. Endovascular treatment of blunt thoracic aortic injury is now widely accepted [14], although its role in penetrating injuries to the chest [15] and blunt or penetrating injury to the abdominal aorta are unclear [16]. In much the same way that increased prehospital tourniquet use has improved outcomes in patients with extremity hemorrhage [3,5,6], effective prehospital control of non-compressible torso hemorrhage could save many lives [17]. Thus, one impetus for the current study is to generate data to inform the development of techniques for control of non-compressible torso hemorrhage that can be used in the pre-operating room phase of care (including the prehospital phase, ED, and non-trauma centers). Additionally, the increasing prevalence of trauma hybrid operating rooms, where open and endovascular techniques can be used as needed [18], will lead to increased and more rapid endovascular treatment options for major vascular injuries. We previously reported anatomic descriptions of non-compressible torso hemorrhage in 543 trauma patients treated at four level 1 trauma centers in Houston, TX and San Antonio, TX between 2008 and 2012 [8], and the current study furthers our understanding in this area. The current study demonstrates that the greatest opportunity for intervention in aortic trauma is patients with penetrating abdominal injury. Of 14 PP deaths, 12 cases (including all 7 who arrived to a trauma center) exsanguinated from gunshot wounds (Table 4 and Table 5). In patients such as these, interventions are needed in the pre-operating room phase of care to extend the period of salvageability and ultimately save lives. These interventions include temporary hemorrhage control by earlier use of established methods such as resuscitative endovascular balloon occlusion of the aorta (REBOA) or novel therapies such as self-expanding intra-abdominal foam [19], hemostatic resuscitation (ideally by whole blood [20]), and faster transport to the operating room for definitive hemorrhage control [17].

The limitations of this study are as follows. Since we only included trauma deaths, we do not capture the total incidence of traumatic aortic injury. Several factors affecting trauma mortality such as distance to major trauma centers at time of injury and other concomitant injuries were not analyzed. One difficulty in translating the success of the military experience to civilian trauma is notification. In the military, injuries usually occur when other team members are immediately available to assist and notify medical personnel. Civilian gunshot trauma, on the other hand, is more often in the context of homicide and suicide where the people involved try to avoid discovery. Indeed, we used time of EMS notification as a surrogate for time of injury (Table 1 and Table 5) since the latter was not known in all cases. Operative reports to review the aortic repair techniques used were not available. We also report a modest incidence of potentially preventable deaths with aortic injury: 14 patients per year, equal to 7% of all deaths with aortic injury (*n* = 192) or 2% of all preventable or potentially preventable deaths in Harris County in 2014 (*n* = 668) [7]. These limitations are counterbalanced by the strengths of this study, which include review of all trauma deaths in the region, the high autopsy rate, and precise anatomic descriptions of aortic injury. When taken in conjunction with our previous work [8], this study better elucidates the scope of the problem as well as potential solutions.

In conclusion, aortic trauma is highly lethal, with 7% of deaths with traumatic aortic injury being potentially preventable and no deaths being preventable. This is in contrast to the 36% preventable/potentially preventable rate in the overall population. Non-preventable deaths were more often a result of blunt trauma and involved the thoracic aorta, whereas potentially preventable deaths were more often a result of penetrating trauma and involved the abdominal aorta. Optimal prehospital and ED treatment include temporizing hemorrhage control, hemostatic resuscitation, and faster transport to definitive treatment.

## Figures and Tables

**Table 1:** Demographics and death data.

	Non-Preventable (*n* = 178)	Potentially Preventable (*n* = 14)	*p*
Age (y)	37 (24, 52)	43 (27, 56)	0.36
Blunt mechanism (*n*, %)	123 (69%)	1 (7%)	<0.01
Death location (*n*, %)			
Scene	149 (84%)	7 (50%)	<0.01 *
Level 1 trauma ctr	14 (8%)	3 (21%)	
ED	9 (64%)	0 (0%)	
ICU	2 (14%)	2 (14%)	
OR	3 (22%)	1 (7%)	
Level 3 trauma ctr ^†^	12 (7%)	4 (29%)	
Level 4 trauma ctr ^†^	1 (<1%)	0 (0%)	
Non-trauma ctr ^†^	2 (1%)	0 (0%)	
Time from EMS notified to death (min)	0 (0, 10)	18 (0, 91)	<0.05
Reason for death (*n*, %) ^‡^			
Hemorrhage	129 (72%)	12 (86%)	0.36
TBI	97 (54%)	2 (14%)	<0.01

EMS, Emergency Medical Service; TBI, traumatic brain injury. * *p* value for scene vs. non-scene death; ^†^ All deaths were in ED; ^‡^ Not mutually exclusive.

**Table 2:** Location of aortic injury by proximal zone.

	Non-Preventable (*n* = 178)	Potentially Preventable (*n* = 14)
Zone 0	46 (26%)	1 (7%)
Zone 1	4 (2%)	0 (0%)
Zone 2	8 (4%)	0 (0%)
Zone 3	29 (16%)	0 (0%)
Zone 4	58 (33%)	1 (7%)
Zone 5	38 (21%)	3 (21%)
Zone 6	7 (4%)	1 (7%)
Zone 7	2 (1%)	0 (0%)
Zone 8	4 (2%)	2 (14%)
Zone 9	6 (3%)	8 (57%)
Multiple injuries	24 (13%)	2 (14%)

**Table 3:** Location of aortic injury by region.

	Non-Preventable (*n* = 178)	Potentially Preventable (*n* = 14)	*p*
Thoracic	166 (93%)	5 (36%)	<0.01
Arch	58 (33%)	1 (7%)	
Descending	140 (79%)	4 (29%)	
Abdominal	22 (37%)	11 (79%)	<0.01
Visceral	15 (8%)	4 (29%)	
Infrarenal	12 (7%)	9 (64%)	
Multiple regions	42 (24%)	4 (29%)	1.00

**Table 4:** Possibly preventable deaths who expired at the scene.

Patient	Zone of Aortic Injury	Injury Mechanism	Cause of Death
Patient 1	5, 9	Motorcycle collision	TBI
Patient 2	6, 7	GSW	Hemorrhage
Patient 3	8, 9	GSW	Hemorrhage
Patient 4	5	GSW	Hemorrhage
Patient 5	9	GSW	Hemorrhage
Patient 6	8	GSW	Hemorrhage
Patient 7	5 to 8	GSW	Hemorrhage and TBI

**Table 5:** Possibly preventable deaths who expired in-hospital. All patients suffered gunshot wounds and died of hemorrhage.

Patient	Zone of Aortic Injury	Location of Death	ISS	Prehospital Interventions	Distance from Scene to Nearest Level 1 Trauma Center (Miles)	Ed SBP	Additional Ed Interventions	Time from Ems Notified to Ed Arrival (min)	Time from Ed Arrival to Death (min)
Patient 8	9	Level 3 trauma center ED	Unknown	CPR, laryngeal airway	20.2	0	None	59	2
Patient 9	9	Level 3 trauma center ED	29	CPR, intubation	16.3	0	Chest tube, blood products, resuscitative thoracotomy	24	23
Patient 10	9	Level 3 trauma center ED	16	CPR, intubation	17.3	0	None	39	1
Patient 11	0	Level 3 trauma center ED	26	CPR, intubation, needle chest thoracostomy	12.3	0	None	23	12
Patient 12	4, 9	Level 1 trauma center OR	75	CPR	17.6	0	Intubation, blood products, resuscitative thoracotomy	31	60
Patient 13	9	Level 1 trauma center ICU	50	CPR	7.8	0	Intubation, blood products, resuscitative thoracotomy	38	511
Patient 14	9	Level 1 trauma center ICU	26	None	12	82	CPR, Intubation, blood products, resuscitative thoracotomy	20	437

ED, Emergency Department; CPR, cardiopulmonary resuscitation; OR, operating room.
